# Survival and Functional Outcomes Following Surgical Resection of Intramedullary Spinal Cord Tumors: A Series of 253 Patients over 22 Years

**DOI:** 10.3390/cancers17132112

**Published:** 2025-06-24

**Authors:** Abdel-Hameed Al-Mistarehi, Khaled J. Zaitoun, Sania Javed, Yuanxuan Xia, Andrew Hersh, Abdul Karim Ghaith, Carly Weber-Levine, Kelly Jiang, Majid Khan, Benjamin Mendelson, Noa Ksabi, Daniel M. Sciubba, Ziya L. Gokaslan, George I. Jallo, Jean-Paul Wolinsky, Nicholas Theodore, Daniel Lubelski

**Affiliations:** 1 Department of Neurosurgery, Johns Hopkins University School of Medicine, Baltimore, MD 21281, USA; aalmist1@jh.edu (A.-H.A.-M.); ahersh2@jhmi.edu (A.H.); aghaith1@jh.edu (A.K.G.); cweberl1@jh.edu (C.W.-L.); kjiang14@jh.edu (K.J.); mkhan9@jhmi.edu (M.K.); gjallo1@jhu.edu (G.I.J.); theodore@jhmi.edu (N.T.); 2 Faculty of Medicine, Jordan University of Science and Technology, Irbid 22110, Jordan; kjzaitoun20@med.just.edu.jo; 3 Department of Medicine, King Edward Medical University, Lahore 54000, Pakistan; sania.javed97@hotmail.com; 4 School of Medicine, West Virginia University, Morgantown, WV 26506, USA; bzm00001@mix.wvu.edu; 5 Department of Neuroscience, Johns Hopkins University, Baltimore, MD 21205, USA; nksabi1@jh.edu; 6 Department of Neurosurgery, Zucker School of Medicine at Hofstra/Northwell Health, Manhasset, NY 11030, USA; dsciubba1@northwell.edu; 7 Department of Neurosurgery, Warren Alpert Medical School, Rhode Island Hospital, Brown University, Providence, RI 02903, USA; ziya_gokaslan@brown.edu; 8 Division of Pediatric Neurosurgery, Institute for Brain Protection Sciences, Johns Hopkins All Children’s Hospital, St. Petersburg, FL 33701, USA; 9 Department of Neurological Surgery, Feinberg School of Medicine, Northwestern University, Chicago, IL 60611, USA; jean-paul.wolinsky@nm.org

**Keywords:** intramedullary spinal cord tumors, IMSCT, ependymoma, astrocytoma, hemangioblastoma, long-term outcomes

## Abstract

**Purpose:** Intramedullary spinal cord tumors (IMSCTs) account for 2–8% of all primary CNS tumors, with ependymal tumors astrocytic tumors and hemangioblastoma being the most prevalent. Due to scarcity of large-scale studies, we aim to provide insights into the long-term neurological and functional outcomes following their resection. **Methods:** A single-center study where retrospective review of all patients’ medical records with IMSCT resection between October 2001 and March 2023 was conducted. Data on demographic characteristics, clinical presentations, and surgical outcomes were collected and analyzed. **Results:** This study included 253 patients (57.7% male) with a mean age of 36.2 ± 19. The cohort comprised ependymal tumors (45.1%), astrocytic tumors (35.6%), hemangioblastoma (11.1%), and miscellaneous tumors (*n* = 21; 8.3%). Differences were observed in age at surgery (*p* < 0.001) and mortality (*p* = 0.002) across tumor types. Gross total resection was more frequently achieved in hemangioblastoma (96.4%) and ependymal tumors (82.5%) compared to astrocytic tumors (55.6%) (*p* < 0.001). Long-term postoperative improvements were significant, with reductions in numbness from 74.7% to 52.2%, pain from 42.2% to 25.7%, and bladder incontinence from 23.7% to 11.6%, particularly in ependymal tumors and astrocytic tumors. Kaplan–Meier analysis showed that patients with ependymal tumors had the highest overall survival rates (94.8% at 5 years, 86.7% at 10 years, 76.3% at 15 years, and 65.4% at 20 years) compared to hemangioblastoma (88.7% at 5 and 10 years, and 53.2% at 15 years) and astrocytic tumors (67.8% at 5 years, 58.1% at 10 and 15 years) (*p* = 0.001). **Conclusions**: This study highlights the differences in survival and long-term functional outcomes among patients with IMSCTs based on tumor histology and grade.

## Simple Summary

Spinal cord tumors that grow inside the cord are rare, and doctors still debate the long-term value of surgery. We reviewed 253 people treated at one hospital over 22 years. The tumors were mainly ependymal tumors (from lining cells), astrocytic tumors (from support cells) and hemangioblastomas (blood-vessel growths). We compared age, surgical success, symptom relief and life span among these groups. Surgeons completely removed almost every hemangioblastoma and most ependymal tumors, but only about half of astrocytic tumors. After surgery many patients felt less numbness and pain, and bladder control improved for some. Twenty years later nearly two-thirds of people with ependymal tumors are still alive, while survival was lower for hemangioblastomas and lowest for astrocytic tumors. Overall, one in three patients improved, one in two stayed the same and fewer than one in four declined. The study shows that the kind of tumor and how fully it can be taken out strongly affects recovery and life expectancy. This knowledge helps doctors set realistic expectations, choose treatments and design follow-up care that protects movement and independence, giving patients and families clearer guidance when facing these challenging tumors.

## 1. Introduction

Intramedullary spinal cord tumors (IMSCTs) account for 2–8% of all primary central nervous system (CNS) tumors [1,2]. There are several histological variants, the most prevalent being ependymoma and astrocytoma, followed by hemangioblastoma [2,3]. Characterized by the invasion and disruption of both white and gray matter, these neoplasms can cause progressive neurological dysfunction, leading to morbidity and mortality and present a formidable challenge in surgical management [4,5]. With advancements in technology—including neuroimaging techniques, microsurgical approaches, and intraoperative neuromonitoring—the management of these tumors has evolved in recent decades, with early surgical intervention becoming the mainstay of treatment in symptomatic patients with IMSCTs [6,7].

However, there is a scarcity of large-scale studies assessing long-term surgical outcomes and rates of permanent neurologic dysfunction. This information is essential to guide patient counseling and identify patients at higher risk of adverse outcomes. The primary objective of this study is to characterize the long-term functional outcomes of IMSCTs and identify relationships between preoperative and perioperative factors and outcomes.

## 2. Materials and Methods

This was a single-center, retrospective cohort study conducted at [blinded for review] and approved by the Institutional Review Board (IRB00378753). A retrospective review of medical records was conducted for patients who underwent surgical resection of an IMSCT between October 2001 and March 2023. Patients were included if they underwent surgical resection of an IMSCT, had histological confirmation of an IMSCT on pathology results, their medical records contained both preoperative and postoperative assessment reports, and had at least one recorded follow-up visit postoperatively.

### 2.1. Study Cohort

For peri-operative and functional outcome analyses (early complications, neurological change, modified McCormick grade), patients were included only if they had both a documented pre-operative examination and postoperative neurological assessment. Patients lacking either time-point were excluded from these specific analyses but were retained in the overall-survival analysis if their date of death or last contact was known.

**Figure 1:**
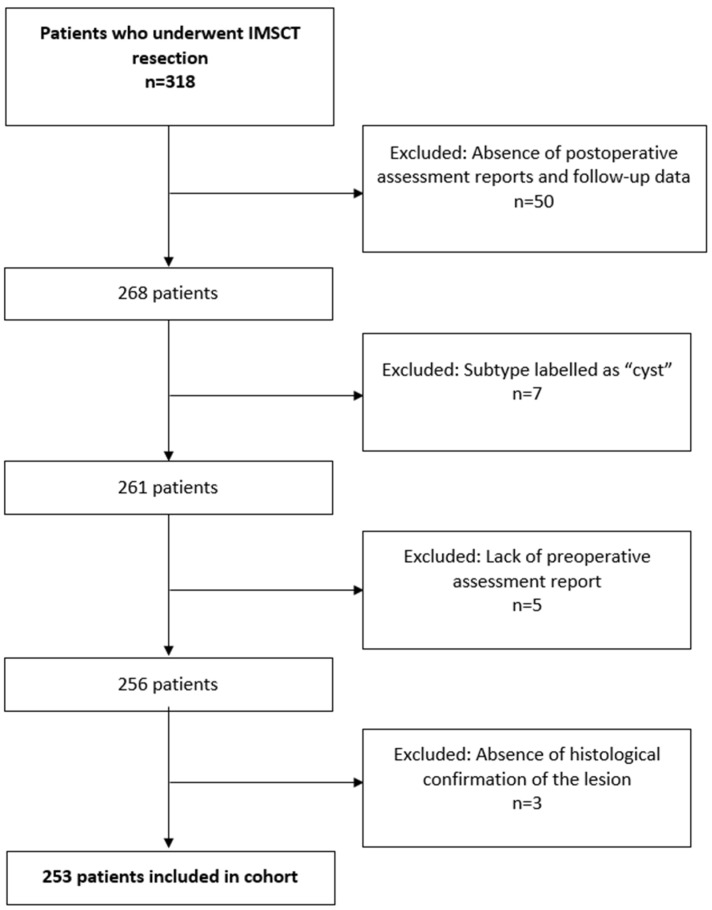
Flow chart of study population.

The initial review of the retrospective database identified 318 patients who underwent IMSCT resection. Of these, 65 patients were excluded due to the absence of postoperative assessment reports and follow-up data (*n* = 50), subtype labeled as “cyst” (*n* = 7), lack of preoperative assessment report (*n* = 5), or absence of histological confirmation of the lesion (*n* = 3). Thus, the final inclusion cohort comprised 253 patients (Figure 1).

### 2.2. Parameters

Patient demographics, clinical status, tumor characteristics, surgical details, and outcomes were collected. Demographics were quantified using a modified Charlson Comorbidity Index (CCI). Tumor data included histology, grade, and location. Surgical features such as resection type, blood loss, and 30-day readmission or reoperation were documented. Gross total resection (GTR) was defined by the complete intraoperative removal of observable tumor tissue, confirmed by operative notes and absence of residual tumor on postoperative imaging within 24–72 h, while en-bloc resection entailed excising the tumor as a single, intact piece without fragmentation [8]. Neurological and functional status, assessed using the modified McCormick Scale (mMS) [6,9], included sensory, motor, and ambulation evaluations. Target outcomes were long-term functional status, overall survival, and tumor recurrence at the last follow-up.

Tumor location and number of involved spinal levels were determined from preoperative MRI imaging. The extent of resection (GTR vs. STR) was defined by the surgeon’s intraoperative assessment and verified by early postoperative MRI (within 24–72 h). All patients with hemangioblastoma tumors were screened for von Hippel-Lindau (vHL) disease.

### 2.3. Statistical Analysis

Data was compiled in Microsoft Excel (Microsoft Corp., Redmond, WA, USA) and analyzed using IBM SPSS Statistics for Windows Version 26.0 (IBM Corp., Armonk, NY, USA). Continuous variables were reported as mean ± standard deviation (SD) and were compared via ANOVA or *t*-test when applicable. Categorical variables were reported as counts and percentages and compared via Fisher’s exact or Chi-squared tests, as appropriate. The McNemar test was used to compare pre- and postoperative neurological and functional status. Kaplan–Meier survival analysis and log-rank tests were used to compare long-term survival across tumor histologies and grades.

## 3. Results

### 3.1. Patient Population

Table 1 summarizes the demographic and clinical features of the study cohort of 253 patients. The overall mean age at the time of surgery was 36.2 ± 19.1 years, with a mean follow-up of 45.3 ± 48.2 months. Patients with ependymal tumors were older, with an average age of 42.0 ± 13.6 years, as compared to those with astrocytic tumors (29.7 ± 23.5 years) (*p* < 0.001). Patients with hemangioblastoma had a mean age of 38.5 ± 15.7 years. The differences in mean age between the three groups were statistically significant (*p* < 0.001). At the same time, there were no significant differences in gender, BMI, and mean follow-up between the three groups of the study cohort.

The proportions of smokers were higher among the patients with hemangioblastoma (14.8%) and ependymal tumors (9.0%) than those with astrocytic tumors (1.3%). Chemotherapy was less frequently utilized in patients with ependymal tumors (12.3%) and hemangioblastoma (7.1%) compared to that in patients with astrocytic tumors (53.3%) (*p* < 0.001). Radiotherapy usage also varied, with 46.7% of patients with astrocytic tumors and 35.7% of those with hemangioblastoma receiving it, compared to 23.7% of patients with ependymal tumors (*p* = 0.003). The maximum radiation dose also showed significant differences, with astrocytic tumors receiving a higher maximum radiation dose when compared to the hemangioblastoma group (4653.5 ± 892.3 cGy vs. 2626.7 ± 948.7 cGy; *p* < 0.001).

The differences in mortality rates between the three groups were statistically significant (*p* = 0.002), with the highest mortality rate among the patients with astrocytic tumors (30.0%), followed by 17.9% with hemangioblastoma and 10.5% among those with ependymal tumors.

Loss or diminution of motor-evoked potentials was observed in ten cases (4.0%), loss of somatosensory-evoked potentials in two (0.8%), global evoked-potential decline in two (0.8%), hemodynamic instability (hypo/hypertension) in two (0.8%), and single instances of hyperthermia, ventilation difficulty, sacrificed dorsal roots, and bradycardia (each 0.4%). Overall, neurophysiologic signal loss (MEPs + SSEPs + global EP decline) constituted 14 of the 20 recorded complications.

### 3.2. Tumor Histology

Table 2 demonstrates the distribution of tumor histologies and WHO grades among the examined cases. WHO grade 2 tumors were predominant, comprising 51.4% of cases. Ependymal tumors were the most frequent pathology (45.1%), primarily consisting of grade 2 tumors. Astrocytic tumors followed at 35.6%, with a more diverse distribution across grades (1–4). Lastly, hemangioblastoma comprised only 11.1% of the cohort.

### 3.3. Tumor Location

The cervical region was the primary site for IMSCTs (37.5%). Significant location differences were noted between ependymal tumors and hemangioblastoma (*p* = 0.046) and between astrocytic tumors and hemangioblastoma (*p* = 0.024). Hemangioblastoma (53.6%) and ependymal tumors (40.4%) were predominantly found in the cervical area, while astrocytic tumors (36.7%) was more common in the cervicothoracic junction. Thoracolumbar or conus lesions (10.3%) were the least common overall (Table 3).

### 3.4. Tumor Resection and Levels Analysis

Table 3 summarizes resection features and outcomes, where en-bloc resection was most commonly achieved in hemangioblastoma (53.6%), followed by ependymal tumors (16.7%), and no en-bloc resections were reported for astrocytic tumors. GTR was achieved in 73.1% of all cases, with a notably higher rate in hemangioblastoma (96.4%) compared to astrocytic tumors (55.6%, *p* < 0.001). Similarly, ependymal tumors (82.5%) also showed a higher rate of GTR compared to astrocytic tumors (55.6%, *p* < 0.001). The mean number of levels affected by the tumor varied significantly among types, with astrocytic tumors affecting more levels compared to ependymal tumors (4.3 ± 2.1 vs. 3.7 ± 2.0, *p* = 0.036), and both spanning more levels compared to hemangioblastoma (2.7 ± 1.4, *p* < 0.001 and *p* = 0.016, respectively).

### 3.5. Indications for 30-Day Readmission and 30-Day Reoperation

Wound complications were the principal drivers of early adverse events: wound dehiscence led to five readmissions (2.0%) and six reoperations (2.4%), while wound infection accounted for four readmissions (1.6%) and three reoperations (1.2%) (Table S1). CSF leakage triggered two readmissions (0.8%) and necessitated a single reoperation (0.4%).

### 3.6. Neurological and Functional Status Presentation Stratified by Tumor Location

Table S2 summarizes neurological and functional status, stratified across different spinal levels of tumor location. Bladder incontinence showed a significant difference between the levels (*p* = 0.019), with 23.7% of the patients reporting symptoms, mainly when lesions were present in the thoracic segment (35.4%). Radicular pain also demonstrated significant variability (*p* = 0.038), affecting 20.6% of the overall cohort, with the highest prevalence in the thoracolumbar or conus segment (38.5%). Additionally, significant differences were observed in the preoperative mMS (*p* = 0.036), with grade I being more common in the cervicothoracic segment (17.9%), while grade V was more common in the thoracic segment (10.4%).

### 3.7. Neurological and Functional Status Change

Table 4 describes how neurological and functional status changed from the preoperative baseline to the long-term postoperative status at the last follow-up. Significant reductions in the proportion of patients experiencing numbness were observed across all groups, with a marked decrease from 74.7% to 52.2% overall.

Bladder incontinence decreased from 23.7% to 11.6% of patients, with the ependymal tumor group dropping from 25.2% to 5.4% (*p* < 0.001). The number of patients with back or neck pain and radicular symptoms also demonstrated significant postoperative reductions overall (*p* < 0.001), especially for those with ependymal tumors. A significant im-provement was observed in ependymal tumors alone (from 59.5% to 41.4%, *p* = 0.007) for motor weakness. The mMS demonstrated significant functional improvements in both ep-endymal tumors and astrocytic tumors. Preoperatively, most patients were mMS Grade II (60.2%), reflecting mild to moderate deficits. Postoperatively, there was a significant in-crease in patients achieving grade I status from 11.2% to 33.3%, indicating minimal or no deficits. This was particularly pronounced in patients with ependymal tumors, where grade I status postoperatively increased to 40.5% from 4.5% preoperatively (*p* < 0.001). Astrocytic tumors also showed notable improvement in grade I status postoperatively, from 18.0% to 30.3%. However, there was also a significant increase in the number of astrocytic tumors patients with grade IV (from 7.9% to 11.2%) and grade V status (from 3.4% to 12.4%) postoperatively (*p* = 0.036). Nevertheless, only 33.3% of patients showed functional improvements, notably in ependymal tumors (45.0%), while the rest had no change or a worsening status. Postoperative local recurrence rates showed variability across histologies (*p* < 0.001), being lowest in ependymal tumors (*n* = 16, 14.4%) and higher in astrocytic tumors (*n* = 39, 43.8%) and hemangioblastoma (*n* = 10, 35.7%). Among the patients with hemangioblastoma, 71.4% were diagnosed with vHL disease, and two patients reported to have multiple hemangioblastoma tumors. During follow-up, five patients (17.9%) died; four of these deaths (80%) occurred in individuals with vHL disease. 

### 3.8. Survival

**Figure 2:**
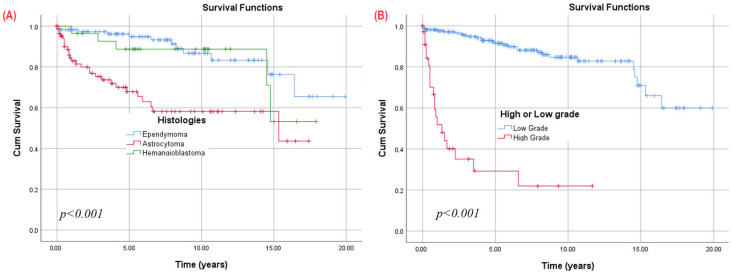
Kaplan–Meier survival analysis of IMSCTs based on (**A**) histology; (**B**) low grade (1–2) vs. high grade (3–4).

Significant differences in survival among the histologies were observed using the Kaplan–Meier analysis (Figure 2A) of postoperative survival for all patients (*n* = 253). Patients with ependymal tumors exhibited significantly (*p* = 0.001) higher overall survival (OS) rates (94.8% survival at 5 years, 86.7% at 10 years, 76.3% at 15 years, and 65.4% at 20 years) compared to those with hemangioblastoma (88.7% survival at 5 and 10 years, 53.2% at 15 years) or astrocytic tumors (67.8% survival at 5 years, 58.1% at 10 and 15 years).

**Figure 3:**
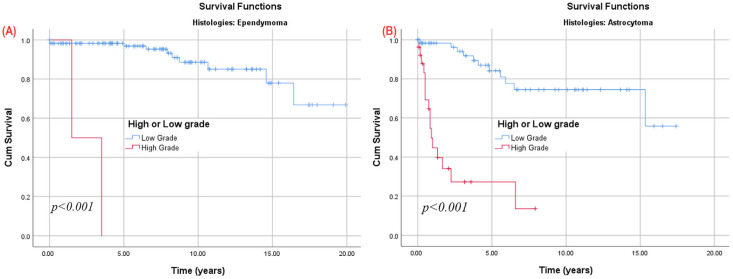
Kaplan–Meier survival analysis of IMSCTs based on low-grade (1–2) vs. high-grade (3–4) tumors for (**A**) EPND and (**B**) ASTR.

Regardless of histology, lesions were categorized into low-grade (1 or 2) and high-grade (3 or 4) groups, with 10-year overall survival (OS) rates of 84.7% for low-grade and 21.9% for high-grade lesions (Figure 2B). This significant survival variance persisted within both the ependymal tumors and ependymal tumors subgroups (Figure 3). Low-grade astrocytic tumors showed survival rates of 84.2% at 5 years and 74.5% at 10 and 15 years, while high-grade (3 or 4) cases had a significantly lower rate of 23.0% at 5 years. For ependymal tumors, low-grade lesions had survival rates of 100.0% at 5 years, 91.5% at 10 years, and 80.5% at 15 years, whereas high-grade (grade 3) cases had a 50.0% survival at 3 years, with none reaching 5 years.

### 3.9. Subgroup Analysis

#### 3.9.1. Astrocytoma Patients

Patients with low-grade spinal astrocytic tumors (Table S3) showed clear relief of sensory and sphincter problems (numbness, bladder, bowel and radicular symptoms all fell, *p* ≤ 0.041), while patients with high-grade ones did not. Motor function declined in both groups, and the modified McCormick scale shifted toward greater disability in high-grade cases (grade V 11.5% to 34.6%), but only marginally improved in low-grade disease. In short, surgery mainly benefits sensory/autonomic symptoms in low-grade tumors, whereas high-grade lesions continued to deteriorate neurologically.

#### 3.9.2. Patients Stratified by Preoperative Modified McCormick Scale (mMS)

Patients with low preoperative mMS (I–II) showed significant improvement in sensory and autonomic symptoms postoperatively—numbness decreased from 74.8% to 51.5%, bladder incontinence from 16.0% to 5.5%, and radicular symptoms from 23.9% to 6.7% (all *p* < 0.05). Muscle weakness worsened slightly from 52.1% to 40.5%, and ambulation dropped from 97.5% to 90.2%. Their functional status remained relatively preserved, with most patients staying within mMS Grades I–II. (Table S4)

Patients with high preoperative mMS (III–V) had limited benefit. Numbness dropped only from 80.0% to 55.4%, and bladder symptoms from 43.1% to 23.1%, while other symptoms showed minimal change. Muscle weakness and poor ambulation persisted at 76.9% and 61.5% postoperatively, and severe disability (mMS IV–V) rose from 29.2% to 43.1%.

#### 3.9.3. Patients with at Least One Year of Follow-Up, Stratified by Tumor Histology

Among 118 patients followed ≥12 months (Table S5), numbness dropped from 74.6% pre-op to 51.7% post-op (*p* < 0.001); reductions were significant in astrocytic tumors (82.0% to 59.0%, *p* = 0.011) and hemangioblastoma (92.3% to 46.2%, *p* = 0.041). Bladder incontinence declined from 21.2% to 6.8% (*p* = 0.003), driven by astrocytic tumors (27.9% to 4.9%, *p* = 0.002). Radicular symptoms fell from 22.0% to 6.8% (*p* = 0.003), and muscle weakness from 57.6% to 42.4% (*p* = 0.014), the latter significant only for astrocytic tumors (65.6% to 39.3%, *p* = 0.003). Ambulatory independence decreased from 96.6% to 88.1% (*p* = 0.009), with ependymal tumors falling from 95.5% to 81.8% (*p* = 0.041). The proportion in modified McCormick Grade I rose from 14.4% to 39.0%, and overall grade distribution improved (*p* < 0.001), most pronounced in astrocytic tumors (*p* < 0.001). No significant changes occurred in bowel dysfunction, back/neck pain, or ambulation for patients with hemangioblastoma.

#### 3.9.4. Patients Stratified by Preoperative Extent of Resection

Table S6 shows that numbness, bladder incontinence, back/neck pain, and radicular symptoms all fell significantly at long-term follow-up (all *p* < 0.001). Improvement in bladder control occurred after GTR from 22.8% to 7.2% (*p* < 0.001) but not STR (*p* = 0.453), and muscle weakness improvement (from 58.7% to 46.1%) was confined to GTR (*p* = 0.016). Ambulatory independence declined modestly overall (*p* = 0.002) from 89.5% to 82.0% without a significant change in STR. The modified McCormick grade distribution shifted toward better function in the entire cohort (*p* < 0.001), and bowel function was unchanged.

## 4. Discussion

This study comprehensively analyzes perioperative characteristics and long-term functional outcomes in 253 patients who underwent surgery for IMSCT. The rarity and complex anatomical location of these tumors pose a significant surgical challenge, and the risk of postoperative neurological deterioration remains a concern [10]. Our study revealed notable variations in patients’ survival and their postoperative neurological and functional outcomes. It was observed that patients with ependymal tumors have better postoperative functional outcomes and better overall survival rates compared to those with astrocytic tumors and hemangioblastoma. Additionally, the high GTR rate in ependymoma and hemangioblastoma correlated with lower recurrence rates and improved long-term neurological function, whereas astrocytic tumor patients experienced higher rates of recurrence and postoperative neurological dysfunction.

Although smoking status differed across histologic groups, there is no evidence that tobacco use plays a causal role in intramedullary astrocytic tumors, ependymal tumors, or hemangioblastoma. We therefore regard the significant *p*-value in Table 1 as a demographic artifact of our younger, predominantly non-smoking astrocytic tumors cohort, not a tumor-specific effect, and did not include smoking in further analyses. Surgical outcomes and preoperative functional status, as measured by the mMS, showed significant differences across tumor types. Overall, most patients presented with preoperative grade 2, and postoperatively, there was a notable increase in the number of patients achieving grade 1 status, particularly in ependymal tumor cases. Previous studies have shown that early surgical intervention increases the possibility of preserving or improving neurological function [6,7,11]. Additionally, the increasing use of intraoperative neurophysiological monitoring has aided surgeons in preserving the neurological function of patients, which could explain our findings. A majority of astrocytic tumor patients, however, showed worsening of mMS postoperatively. Seki et al. reported that 51.5% of the patients with astrocytoma had neurological deterioration postoperatively, and several other studies have reported similar findings [12,13,14]. This can be attributed to the tumor’s infiltrative nature and the high density of eloquent tissue in the spinal cord. Notably, since poor neurological status has been linked to shorter survival, it is crucial to prioritize function over achieving a more extensive resection [15]. Though our study found no significant difference in LOS, discharge dispositions, 30-day readmission, and reoperation rates between the cohorts, in the study conducted by Hersh et al., patients with astrocytoma did exhibit the highest 30-day readmission when compared to ependymoma and hemangioblastoma, underscoring its aggressive nature and the need for more vigilant postoperative monitoring and follow-up care for this group [16].

Symptomatically, our study observed significant postoperative reductions across various parameters and histologies, including the proportions of patients with numbness, bladder incontinence, back or neck pain, and radicular symptoms. There was a notable improvement in motor weakness for patients with ependymal tumors. While the relief of pressure from the tumor on surrounding tissue helps reduce postoperative symptoms, the use of techniques such as SSEPs, MEPs, and EMG during surgery aid surgeons in minimizing neural damage, and the use of microscopes help them to be precise maximizing the chance of a better functional preservation, quicker recovery, and improved quality of life.

Consistent with previous reports, our study also showed ependymal tumors as the predominant histological subtype affecting 45.1% of the cohort, followed by astrocytic tumors (35.6%) and hemangioblastoma (11.1%) [17,18]. Kim et al. reported the mean age of patients with spinal ependymoma as being significantly older (42.1 years) compared to those with astrocytoma (27.6 years), corroborating our findings [19]. Ependymomas are often distinguished by their discrete dissection plane, which makes them amenable to GTR [20]. Notably, the literature suggests that GTR can be achieved in 80–90% of ependymomas [21,22], significantly reducing the necessity for adjuvant therapy, especially in low-grade cases [23,24]. However, the role of adjuvant therapy remains a subject of debate. Some studies advocate for adjuvant radiotherapy or chemotherapy in cases with high-grade tumors or those with STR, while others advocate for a conservative approach [23,25,26,27]. In our study, 82.5% of the ependymal tumor cases underwent GTR, 23.7% of the patients with ependymal tumors received radiotherapy, and even fewer (12.3%) received chemotherapy. Due to mostly being lower grade and having GTR, these tumors are less likely to recur and are associated with a higher overall survival [28]. Indeed, our analysis reveals a favorable survival trend for ependymal tumors.

In contrast, astrocytic tumors presents distinct challenges compared to ependymoma. These tumors also often manifest as low-grade tumors and are less aggressive when compared to primary cranial astrocytoma [29]. In our study, astrocytic tumors exhibited a more heterogeneous distribution across WHO grades, with a majority (70%) classified as low-grade (grades 1 and 2). Additionally, they spanned more spinal levels when compared to other histologies, with an average of 4.3 levels affected, as seen in previous studies [30,31]. Prior studies have consistently underscored the challenge of achieving GTR in cases of spinal astrocytoma due to its infiltrative nature and poorly defined margins [30,31]. Over the years, the literature on the resection of lower-grade astrocytoma has shown that the achievement of GTR varies from as low as 5% to as high as 72.7% of the cases [32]. This increasing trend in GTR rates for low-grade astrocytoma may again be attributed to advancements in microsurgical techniques coupled with the routine use of intraoperative neuromonitoring. In our study, 55.6% of the astrocytic tumor cases underwent GTR. Generally, adjuvant therapy is recommended in cases of incomplete resection, high-grade tumors, and recurrences [33,34]. Given the higher percentage of STR and high-grade astrocytic tumors in our study, it is understandable that 53.3% of the patients received chemotherapy, and 46.7% underwent radiotherapy. Incomplete resection also increases the likelihood of recurrence, contributing to the overall poor prognosis of astrocytoma [35]. In our study, local recurrence was observed in 43.8% of the astrocytic tumor cases. The overall survival was 67.8% at 5 years and 58.1% at 10 years, similar to those reported in the literature, with Khalid et al. reporting overall rates of 64.1% survival at 5 years and 55.0% at 10 years. Nakamura et al. reported 68% at 5 years and only 36% at 10 years [13,34].

Numerous studies indicate that the pathological type and grade of the tumor are the most significant predictors of survival in spinal cord gliomas, with lower grades associated with better overall survival [34,36]. Our study confirms this association as the survival for low-grade astrocytic tumors demonstrated survival rates of 84.2% at 5 years, decreasing to 74.5% at both 10 and 15 years. Similarly, low-grade ependymal tumors showed 100.0% survival at 5 years, 91.5% at 10 years, and 80.5% at 15 years. It is worth noting that none of the tumors underwent molecular analysis or gene mutation testing during the timeframe of the study.

Hemangioblastomas usually have well-defined borders, aiding in GTR, and they are benign WHO grade 1 tumors, derived from primitive embryonic multipotent stem cells called hemangioblastomas, are highly vascular, and typically intra-axial, arising near the pial surface. Extra-axial hemangioblastomas are rarer but can occur. They mostly affect the cerebellum (in more than 80% of the cases), and less commonly the spinal cord (in less than 20% of the cases). Hemangioblastomas of the spinal cord are mainly sporadic and solitary, involving most commonly the thoracic cord (50%), followed by the cervical cord (40%). Younger age of onset and multiple hemangioblastomas should raise the suspicion to the association with vHL syndrome, a rare autosomal dominant familial cancer syndrome [37]. Our study demonstrated a notably high rate of GTR at 96.4%. However, our results indicated that approximately half of the patients with hemangioblastomas underwent en-bloc resection (53.6%) while the remaining underwent intralesional resection (46.4%). This relatively high portion of intralesional resections could be attributed to tumor’s high-volume mass, complex location, or difficulty in separating it from surrounding tissues. While literature indicates that recurrence can occur even with GTR, particularly in cases associated with vHL disease [38,39], the overall reported recurrence rate is typically low. For instance, Yousef et al. reported recurrence in 41.4% of cases, Takeshima et al. reported recurrence in only 3.6% of the cases, while Prokopienko et al. reported no recurrence in their cohort [39,40,41]. However, our study yielded a peculiarly high recurrence rate, with 50% of the cases experiencing local recurrence despite having a high rate of GTR. Among the patients with hemangioblastoma, 71.4% were diagnosed with vHL disease, and all cases of local recurrence occurred in these patients; sporadic tumors showed no recurrence, mirroring prior reports that genuine local regrowth after gross-total resection is rare. The overall survival for our cohort was marginally low when compared to the study conducted by Endo et al., with our cohort having 88.7% survival at 5 years and 10 years, and 53.2% at 15 years and Endo et al. reporting a 5-year survival rate of 96.6%, while Huang et al. reported a similar 5-year survival rate of 87% [42,43]. These comparable survival rates suggest that our findings align with broader trends, although variability in recurrence rates does underscore the need for further investigation into other contributing factors.

Our study revealed that the cervical region was the primary site for spinal tumors, with ependymal tumors and hemangioblastoma being predominantly located in the cervical area, while astrocytic tumors showed a preference for the cervicothoracic junction. This is concordant with the results of previous studies [4,8,44,45]. Tobin et al. suggested that the predilection for the cervical area could be attributed to the higher content of gray matter at that level [44]. The location of the tumor is also an essential determinant in predicting neurological deficits in IMSCT patients. Gembruch et al. reported that motor and sensory deficits were common in patients with tumors in the cervical spine, the pain was often seen in patients with tumors located in the lumbar spine, and sphincter and bladder dysfunction were observed more commonly in thoracic spine tumors [46]. This finding correlated with our study, where 35.4% of the patients with thoracic segment tumors had bladder incontinence, while radicular pain was highest in patients with thoracolumbar or conus tumors (38.5%). In another study, Klekamp reported that patients with thoracic and conus lesions were more likely to have a higher preoperative McCormick grade than the cervical region, which our study confirms [21]. This could be explained by the larger size of the cervical cord, requiring a larger tumor volume to create the mass effect that could lead to apparent neurological deficits.

### Study Limitations

The present study should be viewed in light of certain limitations. First, the retrospective nature of our study introduces selection bias and limits the ability to control confounding variables. The surgeries included were conducted by more than one surgeon, impacting the surgical outcome based on varying experience levels. Additionally, since the study includes data from the last two decades, advancements in surgical techniques, intraoperative monitoring, and postoperative care may have influenced the outcomes, complicating the establishment of standardized comparison. This study was also conducted at a single center, which can potentially introduce institutional bias and thus limit the generalizability of the study results to other healthcare settings. Furthermore, the relatively small sample size and heterogeneity of tumor subtypes may limit the statistical power of our comparisons. In addition, the small proportion of patients who received postoperative irradiation precludes a robust assessment of its impact on progression-free or overall survival. Moreover, among patients with hemangioblastoma, 80% of the deaths occurred in those with VHL disease—a recognized high-risk subgroup—which may overestimate mortality relative to a series composed primarily of sporadic hemangioblastomas. However, despite these limitations, a significant strength of our study lies in having a comparatively larger sample size for these rare pathologies compared to other studies, which helps provide valuable insight and adds to the existing body of knowledge.

## 5. Conclusions

This study provides valuable insights into the perioperative characteristics and long-term outcomes following surgery in patients with IMSCTs. It highlights the interplay of tumor histology, spinal region of the tumor, preoperative neurological status, and extent of resection in determining the neurological and long-term functional outcomes. It was noted that the histological subtype and WHO grade of the tumor were significant predictors of overall survival, with low-grade ependymal tumors having the highest overall survival rates. Histology and tumor location also played a part in determining preoperative symptoms and postoperative neurological status. These findings contribute to the evolving understanding of IMSCTs and can inform evidence-based clinical practices. The results emphasize the importance of the early identification of high-risk patients, targeted interventions, individualized treatment plans, careful preoperative assessments, and advanced microsurgical and monitoring techniques to optimize patient outcomes. Further multicenter prospective studies should aim to validate these findings and explore new strategies for improving the management, prognosis, and quality of life of patients with IMSCTs.

## Figures and Tables

**Table 1:** Demographic, clinical characteristics, and treatment modalities of 253 patients.

	Total (N = 253)	EPND (N = 114)	ASTR (N = 90)	HMNG (N = 28)	MISC (N = 21)	*p*-Value			
						Without MISC	EPND vs. ASTR	EPND vs. HMNG	ASTR vs. HMNG
**Gender, Male**	146.0 (57.7%)	67.0 (58.8%)	54.0 (60.0%)	14.0 (50.0%)	11.0 (52.4%)	0.635 ^1^	0.859 ^1^	0.401 ^1^	0.350 ^1^
** Age at surgery, Years **	36.2 (±19.1)	42.0 (±13.6)	29.7 (±23.5)	38.5 (±15.7)	29.3 (±18.4)	** <0.001 ^2^**	** <0.001 ^4^**	0.236 ^4^	0.066 ^4^
** BMI, kg/m^2^**	27.0 (±10.6)	28.1 (±6.3)	26.4 (±15.8)	26.6 (±7.1)	23.3 (±4.9)	0.614 ^2^	0.374 ^4^	0.315 ^4^	0.973 ^4^
** Smoking status **						** 0.009 ^1^**	** 0.013 ^1^**	0.428 ^1^	** 0.001 ^1^**
Never smoker	166.0 (75.5%)	72.0 (72.0%)	67.0 (89.3%)	16.0 (59.3%)	11.0 (61.1%)				
Current smoker	14.0 (6.4%)	9.0 (9.0%)	1.0 (1.3%)	4.0 (14.8%)	0.0 (0.0%)				
Former smoker	40.0 (18.2%)	19.0 (19.0%)	7.0 (9.3%)	7.0 (25.9%)	7.0 (38.9%)				
Unknown/missing	33	14	15	1	3				
** Charlson comorbidity index **						0.263 ^1^	0.354 ^1^	0.115 ^1^	0.353 ^1^
1	2.0 (0.8%)	2.0 (1.8%)	0.0 (0.0%)	0.0 (0.0%)	0.0 (0.0%)				
2	223.0 (88.1%)	101.0 (88.6%)	81.0 (90.0%)	25.0 (89.3%)	16.0 (76.2%)				
3	12.0 (4.7%)	8.0 (7.0%)	4.0 (4.4%)	0.0 (0.0%)	0.0 (0.0%)				
≥4	16.0 (6.3%)	3.0 (2.6%)	5.0 (5.6%)	3.0 (10.7%)	5.0 (23.8%)				
** Preoperative mMS **						** 0.017 ^1^**	** 0.003 ^1^**	0.686 ^1^	0.147 ^1^
I	30.0 (11.9%)	6.0 (5.3%)	17.0 (18.9%)	3.0 (10.7%)	4.0 (19.0%)				
II	151.0 (59.7%)	79.0 (69.3%)	41.0 (45.6%)	20.0 (71.4%)	11.0 (52.4%)				
III	51.0 (20.2%)	21.0 (18.4%)	22.0 (24.4%)	4.0 (14.3%)	4.0 (19.0%)				
IV	12.0 (4.7%)	4.0 (3.5%)	7.0 (7.8%)	0.0 (0.0%)	1.0 (4.8%)				
V	9.0 (3.6%)	4.0 (3.5%)	3.0 (3.3%)	1.0 (3.6%)	1.0 (4.8%)				
** Duraplasty **	38.0 (15.4%)	16.0 (14.7%)	14.0 (15.9%)	3.0 (10.7%)	5.0 (23.8%)	0.795 ^1^	0.811 ^1^	0.764 ^3^	0.760 ^3^
Unknown/missing	7	5	2	0	0				
** Chemotherapy **	74.0 (29.2%)	14.0 (12.3%)	48.0 (53.3%)	2.0 (7.1%)	10.0 (47.6%)	** <0.001 ^1^**	** <0.001 ^1^**	0.734 ^3^	** <0.001 ^3^**
** Radiotherapy **	92.0 (36.4%)	27.0 (23.7%)	42.0 (46.7%)	10.0 (35.7%)	13.0 (61.9%)	** 0.003 ^1^**	** <0.001 ^1^**	0.194 ^1^	0.308 ^1^
** Max radiotherapy dose ** if used, **Gray**	4214.2 (±1257.7)	4432.5 (±1124.1)	4653.5 (±892.3)	2626.7 (±948.7)	4291.1 (±1456.6)	** <0.001 ^2^**	0.498 ^4^	** <0.001 ^4^**	** <0.001 ^4^**
** Intra-operative complications **	20.0 (8.0%)	12.0 (10.8%)	3.0 (3.4%)	1.0 (3.6%)	4.0 (19.0%)	0.100 ^1^	0.061 ^3^	0.464 ^3^	1.000 ^3^
** Follow-up length, Months **	45.3 (±48.2)	48.4 (±49.6)	42.5 (±48.6)	44.9 (±44.8)	40.4 (±45.0)	0.685 ^2^	0.393 ^4^	0.727 ^4^	0.821 ^4^
** Mortality **	48.0 (19.0%)	12.0 (10.5%)	27.0 (30.0%)	5.0 (17.9%)	4.0 (19.0%)	** 0.002 ^1^**	** <0.001 ^1^**	0.284 ^1^	0.207 ^1^

BMI; Body mass index. ^1^ Chi-squared test. ^2^ Linear model ANOVA. ^3^ Fisher’s exact test. ^4^ Independent samples *t*-test.

**Table 2:** Histologies of studied tumors of 253 patients.

Histology or Classification	N (%)
**Classification**	
WHO grade 1	87.0 (34.4%)
WHO grade 2	130.0 (51.4%)
WHO grade 3	25.0 (9.9%)
WHO grade 4	11.0 (4.3%)
**Histology**	
Ependymal tumors *	114 (45.1%)
WHO grade 1	11.0 (9.6%)
WHO grade 2	101.0 (88.6%)
WHO grade 3	2.0 (1.8%)
**Astrocytic tumors**	90 (35.6%)
WHO grade 1	36.0 (40.0%)
WHO grade 2	27.0 (30.0%)
WHO grade 3	16.0 (17.8%)
WHO grade 4	11.0 (12.2%)
**Hemangioblastoma**	28 (11.1%)
**Miscellaneous**	21 (8.3%)
WHO grade 1	12.0 (57.1%)
WHO grade 2	2.0 (9.5%)
WHO grade 3	7.0 (33.4%)

* Contains two intramedullary myxopapillary ependymomas.

**Table 3:** Surgical overview and outcomes of 253 patients.

	Total (N = 253)	EPND (N = 114)	ASTR (N = 90)	HMNG (N = 28)	MISC (N = 21)	*p*-Value			
						Without MISC	EPND vs. ASTR	EPND vs. HMNG	ASTR vs. HMNG
** Tumor location **						0.069 ^1^	0.437 ^1^	** 0.046 ^1^**	** 0.024 ^1^**
Cervical	95.0 (37.5%)	46.0 (40.4%)	27.0 (30.0%)	15.0 (53.6%)	7.0 (33.3%)				
Cervicothoracic	84.0 (33.2%)	39.0 (34.2%)	33.0 (36.7%)	3.0 (10.7%)	9.0 (42.9%)				
Thoracic	48.0 (19.0%)	21.0 (18.4%)	21.0 (23.3%)	5.0 (17.9%)	1.0 (4.8%)				
Thoracolumbar/conus	26.0 (10.3%)	8.0 (7.0%)	9.0 (10.0%)	5.0 (17.9%)	4.0 (19.0%)				
** Type of Resection **						** <0.001 ^1^**	** <0.001 ^3^**	** <0.001 ^1^**	** <0.001 ^3^**
En-bloc	34.0 (13.4%)	19.0 (16.7%)	0.0 (0.0%)	15.0 (53.6%)	0.0 (0.0%)				
Intralesional	219.0 (86.6%)	95.0 (83.3%)	90.0 (100.0%)	13.0 (46.4%)	21.0 (100.0%)				
** Extent of resection **						** <0.001 ^1^**	** <0.001 ^1^**	0.076 ^3^	** <0.001 ^3^**
GTR	185.0 (73.1%)	94.0 (82.5%)	50.0 (55.6%)	27.0 (96.4%)	14.0 (66.7%)				
STR	68.0 (26.9%)	20.0 (17.5%)	40.0 (44.4%)	1.0 (3.6%)	7.0 (33.3%)				
** Tumor levels span **	3.8 (±2.0)	3.7 (±2.0)	4.3 (±2.1)	2.7 (±1.4)	3.7 (±1.2)	** <0.001 ^2^**	** 0.036 ^4^**	** 0.016 ^4^**	** <0.001 ^4^**
** Blood transfusion during surgery **	10.0 (6.3%)	5.0 (7.1%)	2.0 (3.5%)	2.0 (13.3%)	1.0 (6.2%)	0.353 ^1^	0.458 ^3^	0.602 ^3^	0.189 ^3^
Unknown/missing	95	44	33	13	5				
** Surgery duration, mins **	270.3 (±103.0)	287.1 (±121.7)	249.0 (±94.8)	300.1 (±75.7)	234.5 (±46.6)	0.141 ^2^	0.109 ^4^	0.689 ^4^	0.059 ^4^
** Estimated blood loss during surgery, mL **	188.0 (±393.1)	230.4 (±461.6)	152.9 (±401.6)	191.3 (±147.9)	138.8 (±227.5)	0.529 ^2^	0.291 ^4^	0.691 ^4^	0.656 ^4^
** Length of stay, Days **	8.0 (±8.3)	8.0 (±7.7)	7.6 (±8.5)	9.4 (±10.5)	7.9 (±7.4)	0.628 ^2^	0.749 ^4^	0.431 ^4^	0.370 ^4^
** Hospital discharges **									
Home	114.0 (45.1%)	50.0 (43.9%)	38.0 (42.2%)	17.0 (60.7%)	9.0 (42.9%)	0.210 ^1^	0.815 ^1^	0.109 ^1^	0.087 ^1^
Subacute rehab	20.0 (7.9%)	7.0 (6.1%)	12.0 (13.3%)	1.0 (3.6%)	0.0 (0.0%)	0.115 ^1^	0.079 ^1^	1.000 ^3^	0.297 ^3^
ACIR	116.0 (45.8%)	55.0 (48.2%)	40.0 (44.4%)	10.0 (35.7%)	11.0 (52.4%)	0.481 ^1^	0.589 ^1^	0.233 ^1^	0.414 ^1^
Hospice care	1.0 (0.4%)	0.0 (0.0%)	0.0 (0.0%)	0.0 (0.0%)	1.0 (4.8%)	-	-	-	-
Death	2.0 (0.8%)	2.0 (1.8%)	0.0 (0.0%)	0.0 (0.0%)	0.0 (0.0%)	0.3521	0.5043	1.0003	-
** Readmission within 30 days **	27.0 (10.7%)	8.0 (7.0%)	12.0 (13.3%)	1.0 (3.6%)	6.0 (28.6%)	0.165 ^1^	0.132 ^1^	0.689 ^3^	0.297 ^3^
** Reoperation within 30 days **	16.0 (6.3%)	7.0 (6.1%)	6.0 (6.7%)	1.0 (3.6%)	2.0 (9.5%)	0.833 ^1^	0.879 ^1^	1.000 ^3^	1.000 ^3^

ACIR; Acute Comprehensive Inpatient Rehab, GTR; Gross Total Resection, mMS; modified McCormick Scale, STR; Subtotal Resection. ^1^ Chi-squared test. ^2^ Linear Model ANOVA. ^3^ Fisher’s exact test. ^4^ Independent samples *t*-test.

**Table 4:** Preoperative and long-term postoperative outcomes, neurological, and functional status of 249 patients.

	Total (N = 249)	EPND (N = 111)	ASTR (N = 89)	HMNG (N = 28)	MISC (N = 21)	*p*-Value			
						Without MISC	EPND	ASTR	HMNG
** Numbness **						** <0.001 ^1^**	** <0.001 ^1^**	** <0.001 ^1^**	** 0.021 ^1^**
Preoperative	186.0 (74.7%)	90.0 (81.1%)	60.0 (67.4%)	24.0 (85.7%)	12.0 (57.1%)				
Postoperative	130.0 (52.2%)	66.0 (59.5%)	38.0 (42.7%)	16.0 (57.1%)	10.0 (47.6%)				
** Bladder incontinence **						** <0.001 ^1^**	** <0.001 ^1^**	0.210 ^1^	1.000 ^1^
Preoperative	59.0 (23.7%)	28.0 (25.2%)	23.0 (25.8%)	3.0 (10.7%)	5.0 (23.8%)				
Postoperative	29.0 (11.6%)	6.0 (5.4%)	16.0 (18.0%)	2.0 (7.1%)	5.0 (23.8%)				
** Bowel dysfunctions **						1.000 ^1^	0.219 ^1^	1.000 ^1^	1.000 ^1^
Preoperative	22.0 (8.8%)	5.0 (4.5%)	12.0 (13.5%)	2.0 (7.1%)	3.0 (14.3%)				
Postoperative	21.0 (8.4%)	1.0 (0.9%)	12.0 (13.5%)	2.0 (7.1%)	6.0 (28.6%)				
** Back/neck pain **						** <0.001 ^1^**	** <0.001 ^1^**	0.291 ^1^	0.092 ^1^
Preoperative	105.0 (42.2%)	48.0 (43.2%)	38.0 (42.7%)	11.0 (39.3%)	8.0 (38.1%)				
Postoperative	64.0 (25.7%)	22.0 (19.8%)	30.0 (33.7%)	4.0 (14.3%)	8.0 (38.1%)				
** Radicular symptoms **						** <0.001 ^1^**	** <0.001 ^1^**	** 0.035 ^1^**	0.219 ^1^
Preoperative	51.0 (20.5%)	31.0 (27.9%)	13.0 (14.6%)	5.0 (17.9%)	2.0 (9.5%)				
Postoperative	16.0 (6.4%)	8.0 (7.2%)	4.0 (4.5%)	1.0 (3.6%)	3.0 (14.3%)				
** Muscle weakness **						0.175 ^1^	** 0.007 ^1^**	0.170 ^1^	0.065 ^1^
Preoperative	144.0 (57.8%)	66.0 (59.5%)	50.0 (56.2%)	19.0 (67.9%)	9.0 (42.9%)				
Postoperative	130.0 (52.2%)	46.0 (41.4%)	58.0 (65.2%)	12.0 (42.9%)	14.0 (66.7%)				
** Ambulation ability **						** <0.001 ^1^**	0.180 ^1^	** 0.013 ^1^**	1.000 ^1^
Preoperative	222.0 (89.2%)	104.0 (93.7%)	74.0 (83.1%)	26.0 (92.9%)	18.0 (85.7%)				
Postoperative	202.0 (81.1%)	99.0 (89.2%)	63.0 (70.8%)	25.0 (89.3%)	15.0 (71.4%)				
** modified McCormick Scale (mMS) **									
Preoperative									
I	28.0 (11.2%)	5.0 (4.5%)	16.0 (18.0%)	3.0 (10.7%)	4.0 (19.0%)				
II	150.0 (60.2%)	78.0 (70.3%)	41.0 (46.1%)	20.0 (71.4%)	11.0 (52.4%)				
III	50.0 (20.1%)	20.0 (18.0%)	22.0 (24.7%)	4.0 (14.3%)	4.0 (19.0%)				
IV	12.0 (4.8%)	4.0 (3.6%)	7.0 (7.9%)	0.0 (0.0%)	1.0 (4.8%)				
V	9.0 (3.6%)	4.0 (3.6%)	3.0 (3.4%)	1.0 (3.6%)	1.0 (4.8%)				
Postoperative						** <0.001 ^1^**	** <0.001 ^1^**	** 0.036 ^1^**	0.300 ^1^
I	83.0 (33.3%)	45.0 (40.5%)	27.0 (30.3%)	7.0 (25.0%)	4.0 (19.0%)				
II	79.0 (31.7%)	34.0 (30.6%)	24.0 (27.0%)	14.0 (50.0%)	7.0 (33.3%)				
III	53.0 (21.3%)	26.0 (23.4%)	17.0 (19.1%)	6.0 (21.4%)	4.0 (19.0%)				
IV	19.0 (7.6%)	3.0 (2.7%)	10.0 (11.2%)	0.0 (0.0%)	6.0 (28.6%)				
V	15.0 (6.0%)	3.0 (2.7%)	11.0 (12.4%)	1.0 (3.6%)	0.0 (0.0%)				
** Change **						**0.030 ^2^**	-	-	-
Worse (grade or more)	59.0 (23.7%)	21.0 (18.9%)	26.0 (29.2%)	5.0 (17.9%)	7.0 (33.3%)				
Same	107.0 (43.0%)	40.0 (36.0%)	39.0 (43.8%)	16.0 (57.1%)	12.0 (57.1%)				
Improved (grade or more)	83.0 (33.3%)	50.0 (45.0%)	24.0 (27.0%)	7.0 (25.0%)	2.0 (9.5%)				
** Local recurrence of the tumor **	79.0 (31.7%)	16.0 (14.4%)	39.0 (43.8%)	10.0 (35.7%)	10.0 (47.6%)	** <0.001 ^2^**	-	-	-

^1^ McNemar test. ^2^ Chi-squared test.

## Data Availability

The datasets generated and analyzed during the current study are available with the corresponding author upon request.

## Supplementary Materials

The following supporting information can be downloaded at: https://www.mdpi.com/article/10.3390/cancers17132112/s1, Table S1: Indications for 30-Day Readmission and 30-Day Reoperation; Table S2: Tumor Grading, Neurological and Functional Status Presentation Stratified by spinal region of 253 Patients; Table S3: Preoperative and Long-Term Postoperative Outcomes, Neurological, and Functional Status for Astrocytoma tumors (*n* = 89); Table S4: Preoperative and Long-Term Postoperative Outcomes, Neurological, and Functional Status Stratified by McCormick Scale without MISC group (*n* = 228); Table S5: Preoperative and Long-Term Postoperative Outcomes, Neurological, and Functional Status Stratified by Histology For More Than 1 Year of follow-up (*n* = 118); Table S6: Preoperative and Long-Term Postoperative Outcomes, Neurological, and Functional Status Stratified by Extent of Resection without MISC group (*n* = 228).

## Abbreviations

IMSCT	intramedullary spinal cord tumor
CNS	central nervous system
BMI	body mass index
CCI	Charlson Comorbidity Index
CNS	central nervous system
EBL	estimated blood loss
GTR	gross total resection
STR	sub-total resection
LOS	length of stay
mMS	modified McCormick Scale
OS	overall survival
STR	subtotal resection
EPND	ependymoma
ASTR	astrocytoma
HMNG	hemangioblastoma
MISC	miscellaneous
