# Supplementary material for: Survival and Functional Outcomes Following Surgical Resection of Intramedullary Spinal Cord Tumors: A Series of 253 Patients over 22 Years
Source: Cancers (Basel). 2025 Jun 24;17(13):2112. doi: 10.3390/cancers17132112 (PMC12248634; doi:10.3390/cancers17132112)
Supplement: Supplementary file 1 [file cancers-17-02112-s001.zip › cancers-3577573-supplementary.pdf]

Table S1. *Indications for 30-Day Readmission and 30-Day Reoperation.*

Complication	Readmissions (N=27)	Reoperations (N=16)
Wound dehiscence	5 (2%)	6 (2.4%)
Wound infection	4 (1.6%)	3 (1.2%)
Hematoma	2 (0.8%)	3 (1.2%)
CSF leak	2 (0.8%)	1 (0.4%)
Rehabilitation admission	2 (0.8%)	0 (0%)
Pneumonia	2 (0.8%)	0 (0%)
Post operative fever	2 (0.8%)	0 (0%)
Headache with increased CSF/ICP	1 (0.4%)	1 (0.4%)
Planned further excision	1 (0.4%)	1 (0.4%)
Acute motor strength loss	1 (0.4%)	1 (0.4%)
Neurologic weakness/fall	1 (0.4%)	0 (0%)
Urinary tract infection	1 (0.4%)	0 (0%)
Altered mentation	1 (0.4%)	0 (0%)
Pulmonary embolism	1 (0.4%)	0 (0%)
Retinal angiomas (VHL)	1 (0.4%)	0 (0%)

Table S2. *Tumor Grading, Neurological and Functional Status Presentation Stratified by spinal region of 253 Patients.*

	Total (N=253)	Cervical (N=95)	Cervicothoracic (N=84)	Thoracic (N=48)	Thoracolumbar/conus (N=26)	p value
**Numbness**	162.0 (64.0%)	61.0 (64.2%)	51.0 (60.7%)	32.0 (66.7%)	18.0 (69.2%)	0.837[1]
**Paresthesia**	123.0 (48.6%)	52.0 (54.7%)	39.0 (46.4%)	17.0 (35.4%)	15.0 (57.7%)	0.122[1]
**Bladder incontinence**	60.0 (23.7%)	13.0 (13.7%)	22.0 (26.2%)	17.0 (35.4%)	8.0 (30.8%)	**0.019**[1]
**Bowel dysfunctions**	23.0 (9.1%)	5.0 (5.3%)	8.0 (9.5%)	6.0 (12.5%)	4.0 (15.4%)	0.305[1]
**Back and/or neck pain**	107.0 (42.3%)	44.0 (46.3%)	34.0 (40.5%)	17.0 (35.4%)	12.0 (46.2%)	0.608[1]
**Radicular pain**	52.0 (20.6%)	13.0 (13.7%)	17.0 (20.2%)	12.0 (25.0%)	10.0 (38.5%)	**0.038**[1]
**Muscle weakness**	145.0 (57.3%)	59.0 (62.1%)	43.0 (51.2%)	25.0 (52.1%)	18.0 (69.2%)	0.238[1]
**Ambulation ability**						0.081[1]
Not ambulatory	27.0 (10.7%)	5.0 (5.3%)	9.0 (10.7%)	8.0 (16.7%)	5.0 (19.2%)	
Ambulatory	226.0 (89.3%)	90.0 (94.7%)	75.0 (89.3%)	40.0 (83.3%)	21.0 (80.8%)	
**Preoperative mMS**						**0.036**[1]
I	30.0 (11.9%)	9.0 (9.5%)	15.0 (17.9%)	4.0 (8.3%)	2.0 (7.7%)	
II	151.0 (59.7%)	64.0 (67.4%)	48.0 (57.1%)	26.0 (54.2%)	13.0 (50.0%)	
III	51.0 (20.2%)	18.0 (18.9%)	17.0 (20.2%)	9.0 (18.8%)	7.0 (26.9%)	
IV	12.0 (4.7%)	4.0 (4.2%)	1.0 (1.2%)	4.0 (8.3%)	3.0 (11.5%)	
V	9.0 (3.6%)	0.0 (0.0%)	3.0 (3.6%)	5.0 (10.4%)	1.0 (3.8%)	
**Classification**						0.187[1]
WHO grade I	87.0 (34.4%)	35.0 (36.8%)	23.0 (27.4%)	16.0 (33.3%)	13.0 (50.0%)	
WHO grade II	130.0 (51.4%)	49.0 (51.6%)	50.0 (59.5%)	25.0 (52.1%)	6.0 (23.1%)	
WHO grade III	25.0 (9.9%)	8.0 (8.4%)	8.0 (9.5%)	4.0 (8.3%)	5.0 (19.2%)	
WHO grade IV	11.0 (4.3%)	3.0 (3.2%)	3.0 (3.6%)	3.0 (6.2%)	2.0 (7.7%)	

mMS; modified McCormick Scale.

1. Chi-squared test

Table S3. *Preoperative and Long-Term Postoperative Outcomes, Neurological, and Functional Status for Astrocytoma tumors (n=89).*

**Symptom**		**Total (N = 89)**	**High-grade (N = 26)**	**Low-grade (N = 63)**	**p-value High**	**p-value Low**
**Numbness**	Pre	60 (67.4%)	20 (76.9%)	40 (63.5%)	0.096	**0.005**
	Post	38 (42.7%)	13 (50.0%)	25 (39.7%)		
**Bladder incontinence**	Pre	23 (25.8%)	10 (38.5%)	13 (20.6%)	0.724	**0.039**
	Post	16 (18.0%)	12 (46.2%)	4 (6.3%)		
**Bowel dysfunction**	Pre	12 (13.5%)	5 (19.2%)	7 (11.1%)	0.077	**0.041**
	Post	12 (13.5%)	11 (42.3%)	1 (1.6%)		
**Back/neck pain**	Pre	38 (42.7%)	15 (57.7%)	23 (36.5%)	0.789	0.361
	Post	30 (33.7%)	13 (50.0%)	17 (27.0%)		
**Radicular symptoms**	Pre	13 (14.6%)	3 (11.5%)	10 (15.9%)	1.000	**0.027**
	Post	4 (4.5%)	2 (7.7%)	2 (3.2%)		
**Muscle weakness**	Pre	50 (56.2%)	22 (84.6%)	28 (44.4%)	0.617	0.286
	Post	58 (65.2%)	24 (92.3%)	34 (54.0%)		
**Ambulation ability**	Pre	74 (83.1%)	14 (53.8%)	60 (95.2%)	0.343	**0.023**
	Post	63 (70.8%)	10 (38.5%)	53 (84.1%)		
**modified McCormick Scale (mMS)**	Pre – I	16 (18.0%)	0 (0.0%)	16 (25.4%)	**0.046**	0.112
	Pre – II	41 (46.1%)	10 (38.5%)	31 (49.2%)		
	Pre – III	22 (24.7%)	8 (30.8%)	14 (22.2%)		
	Pre – IV	7 (7.9%)	5 (19.2%)	2 (3.2%)		
	Pre – V	3 (3.4%)	3 (11.5%)	0 (0.0%)		
	Post – I	27 (30.3%)	2 (7.7%)	25 (39.7%)		
	Post – II	24 (27.0%)	2 (7.7%)	22 (34.9%)		
	Post – III	17 (19.1%)	7 (26.9%)	10 (15.9%)		
	Post – IV	10 (11.2%)	6 (23.1%)	4 (6.3%)		
	Post – V	11 (12.4%)	9 (34.6%)	2 (3.2%)		

Table S4. *Preoperative and Long-Term Postoperative Outcomes, Neurological, and Functional Status Stratified by McCormick Scale without MISC group (n=228).*

		Total (N = 228)	High mMS (N = 65)	Low mMS (N = 163)	p-value High	p-value Low
**Numbness**	Pre	174 (76.3%)	52 (80.0%)	122 (74.8%)	**0.006**	**<0.001**
	Post	120 (52.6%)	36 (55.4%)	84 (51.5%)		
**Bladder incontinence**	Pre	54 (23.7%)	28 (43.1%)	26 (16.0%)	**0.026**	**0.002**
	Post	24 (10.5%)	15 (23.1%)	9 (5.5%)		
**Bowel dysfunction**	Pre	19 (8.3%)	8 (12.3%)	11 (6.7%)	0.752	0.181
	Post	15 (6.6%)	10 (15.4%)	5 (3.1%)		
**Back/neck pain**	Pre	97 (42.5%)	28 (43.1%)	69 (42.3%)	**0.038**	**0.002**
	Post	56 (24.6%)	16 (24.6%)	40 (24.5%)		
**Radicular symptoms**	Pre	49 (21.5%)	10 (15.4%)	39 (23.9%)	0.043	**<0.001**
	Post	13 (5.7%)	2 (3.1%)	11 (6.7%)		
**Muscle weakness**	Pre	135 (59.2%)	50 (76.9%)	85 (52.1%)	1	**0.023**
	Post	116 (50.9%)	50 (76.9%)	66 (40.5%)		
**Ambulation ability**	Pre	204 (89.5%)	45 (69.2%)	159 (97.5%)	0.228	**0.006**
	Post	187 (82.0%)	40 (61.5%)	147 (90.2%)		
**modified McCormick Scale (mMS)**	Pre – I	24 (10.5%)	0 (0.0%)	24 (14.7%)	**<0.001**	**<0.001**
	Pre – II	139 (61.0%)	0 (0.0%)	139 (85.3%)		
	Pre – III	46 (20.2%)	46 (70.8%)	0 (0.0%)		
	Pre – IV	11 (4.8%)	11 (16.9%)	0 (0.0%)		
	Pre – V	8 (3.5%)	8 (12.3%)	0 (0.0%)		
	Post – I	79 (34.6%)	9 (13.8%)	70 (42.9%)		
	Post – II	72 (31.6%)	15 (23.1%)	57 (35.0%)		
	Post – III	49 (21.5%)	22 (33.8%)	27 (16.6%)		
	Post – IV	13 (5.7%)	8 (12.3%)	5 (3.1%)		
	Post – V	15 (6.6%)	11 (16.9%)	4 (2.5%)		

Table S5. *Preoperative and Long-Term Postoperative Outcomes, Neurological, and Functional Status Stratified by Histology For More Than 1 Year of follow-up (n=118).*

		Total (N = 118)	ASTR (N = 61)	EPND (N = 44)	HMNG (N = 13)	p-value Total	p-value ASTR	p-value EPND	p-value HMNG
**Numbness**	Pre	88 (74.6%)	50 (82.0%)	26 (59.1%)	12 (92.3%)	**<0.001**	**0.011**	0.146	**0.041**
	Post	61 (51.7%)	36 (59.0%)	19 (43.2%)	6 (46.2%)				
**Bladder incontinence**	Pre	25 (21.2%)	17 (27.9%)	7 (15.9%)	1 (7.7%)	**0.003**	**0.002**	0.752	1
	Post	8 (6.8%)	3 (4.9%)	5 (11.4%)	0 (0.0%)				
**Bowel dysfunction**	Pre	8 (6.8%)	4 (6.6%)	2 (4.5%)	2 (15.4%)	0.114	0.134	1	0.48
	Post	2 (1.7%)	0 (0.0%)	2 (4.5%)	0 (0.0%)				
**Back/neck pain**	Pre	43 (36.4%)	24 (39.3%)	15 (34.1%)	4 (30.8%)	0.086	0.078	1	0.617
	Post	30 (25.4%)	14 (23.0%)	14 (31.8%)	2 (15.4%)				
**Radicular symptoms**	Pre	26 (22.0%)	18 (29.5%)	7 (15.9%)	1 (7.7%)	**0.003**	**0.012**	0.289	1
	Post	8 (6.8%)	5 (8.2%)	3 (6.8%)	0 (0.0%)				
**Muscle weakness**	Pre	68 (57.6%)	40 (65.6%)	20 (45.5%)	8 (61.5%)	**0.014**	**0.003**	1	0.371
	Post	50 (42.4%)	24 (39.3%)	21 (47.7%)	5 (38.5%)				
**Ambulation ability**	Pre	114 (96.6%)	59 (96.7%)	42 (95.5%)	13 (100.0%)	**0.009**	0.221	**0.041**	NA
	Post	104 (88.1%)	55 (90.2%)	36 (81.8%)	13 (100.0%)				
**modified McCormick Scale (mMS)**	Pre – I	17 (14.4%)	4 (6.6%)	11 (25.0%)	2 (15.4%)	**<0.001**	**<0.001**	0.099	0.955
	Pre – II	76 (64.4%)	44 (72.1%)	23 (52.3%)	9 (69.2%)				
	Pre – III	21 (17.8%)	10 (16.4%)	9 (20.5%)	2 (15.4%)				
	Pre – IV	2 (1.7%)	1 (1.6%)	1 (2.3%)	0 (0.0%)				
	Pre – V	2 (1.7%)	2 (3.3%)	0 (0.0%)	0 (0.0%)				
	Post – I	46 (39.0%)	24 (39.3%)	19 (43.2%)	3 (23.1%)				
	Post – II	41 (34.7%)	20 (32.8%)	14 (31.8%)	7 (53.8%)				
	Post – III	24 (20.3%)	15 (24.6%)	6 (13.6%)	3 (23.1%)				
	Post – IV	5 (4.2%)	2 (3.3%)	3 (6.8%)	0 (0.0%)				
	Post – V	2 (1.7%)	0 (0.0%)	2 (4.5%)	0 (0.0%)				

Table S6. *Preoperative and Long-Term Postoperative Outcomes, Neurological, and Functional Status Stratified by Extent of Resection without MISC group (n=228).*

		Total (N = 228)	GTR (N = 167)	STR (N = 61)	p-value Total	p-value GTR	p-value STR
**Numbness**	Pre	174 (76.3%)	124 (74.3%)	50 (82.0%)	**<0.001**	**<0.001**	**<0.001**
	Post	120 (52.6%)	89 (53.3%)	31 (50.8%)			
**Bladder incontinence**	Pre	54 (23.7%)	38 (22.8%)	16 (26.2%)	**<0.001**	**<0.001**	0.453
	Post	24 (10.5%)	12 (7.2%)	12 (19.7%)			
**Bowel dysfunction**	Pre	19 (8.3%)	12 (7.2%)	7 (11.5%)	0.540	0.332	1
	Post	15 (6.6%)	7 (4.2%)	8 (13.1%)			
**Back/neck pain**	Pre	97 (42.5%)	68 (40.7%)	29 (47.5%)	**<0.001**	**<0.001**	0.265
	Post	56 (24.6%)	34 (20.4%)	22 (36.1%)			
**Radicular symptoms**	Pre	49 (21.5%)	39 (23.4%)	10 (16.4%)	**<0.001**	**<0.001**	**0.043**
	Post	13 (5.7%)	11 (6.6%)	2 (3.3%)			
**Muscle weakness**	Pre	135 (59.2%)	98 (58.7%)	37 (60.7%)	0.054	**0.016**	0.814
	Post	116 (50.9%)	77 (46.1%)	39 (63.9%)			
**Ambulation ability**	Pre	204 (89.5%)	154 (92.2%)	50 (82.0%)	**0.002**	**0.010**	0.149
	Post	187 (82.0%)	143 (85.6%)	44 (72.1%)			
**modified McCormick Scale (mMS)**	Pre – I	24 (10.5%)	18 (10.8%)	6 (9.8%)	**<0.001**	**<0.001**	**0.016**
	Pre – II	139 (61.0%)	108 (64.7%)	31 (50.8%)			
	Pre – III	46 (20.2%)	29 (17.4%)	17 (27.9%)			
	Pre – IV	11 (4.8%)	6 (3.6%)	5 (8.2%)			
	Pre – V	8 (3.5%)	6 (3.6%)	2 (3.3%)			
	Post – I	79 (34.6%)	65 (38.9%)	14 (23.0%)			
	Post – II	72 (31.6%)	56 (33.5%)	16 (26.2%)			
	Post – III	49 (21.5%)	32 (19.2%)	17 (27.9%)			
	Post – IV	13 (5.7%)	5 (3.0%)	8 (13.1%)			
	Post – V	15 (6.6%)	9 (5.4%)	6 (9.8%)			
